# Significant increases in Donghong kiwifruit yield by a novel umbrella-shaped trellis system and identification of associated molecular mechanisms

**DOI:** 10.3389/fpls.2023.1143525

**Published:** 2023-03-13

**Authors:** Honghong Deng, Yao Li, Changqing Pang, Kun Zhang, Xinbo Tian, Tong Wang, Yan Liang, Zunzhen He, Yuxuan Lang, Jinbao Fang, Lijin Lin, Jin Wang, Xiulan Lv, Hui Xia, Dong Liang

**Affiliations:** ^1^ Institute of Pomology and Olericulture, College of Horticulture, Sichuan Agricultural University, Chengdu, China; ^2^ Zhengzhou Fruit Research Institute, Chinese Academy of Agricultural Sciences, Zhengzhou, China

**Keywords:** budbreak, carbon to nitrogen ratio, endogenous hormones, fruiting cane, gas exchange parameters

## Abstract

China is the largest kiwifruit producer in the world, accounting for more than half of the total. However, in terms of yield per unit area, China is much lower than the global average and lags behind that of other countries. Yield improvement is of critical importance for the current kiwifruit industry in China. In this study, an improved overhead pergolas trellis (OPT) system, namely, the umbrella-shaped trellis (UST) system, was developed for Donghong kiwifruit, which is now the second most popular and widely cultivated red-fleshed kiwifruit in China. Surprisingly, the estimated yield on the UST system was more than two times higher than that with a traditional OPT, while the external fruit quality was maintained and the internal fruit quality was improved. One of the mechanisms contributing to the yield improvement was the significant promotion of the vegetative growth of canes at 6 ~ 10 mm in diameter by the UST system. The upper canopy of the UST treatment served as a natural shading condition for the lower fruiting canopy and thus had positive effects on the accumulation of chlorophylls and total carotenoids in the fruiting canopy. The most productive zones on the fruiting canes (6 ~ 10 mm in diameter) contained significantly higher (*P* < 0.05) levels of zeatin riboside (ZR) and auxin (IAA) and ratios of ZR/gibberellin (GA), ZR/abscisic acid (ABA), and ABA/GA. A relatively high carbon/nitrogen ratio may promote the flower bud differentiation process of Donghong kiwifruit. The outcomes of this study provide a scientific basis for manifold increase in production of kiwifruit and contribute to the sustainability of the kiwifruit industry.

## Introduction

Kiwi fruit is the berry of a woody deciduous climbing vine species belonging to the genus *Actinidia* Lindl. of the family Actinidiaceae (Ferguson, 2016). It has gained enormous popularity in recent decades due to its exceptionally high levels of vitamin C and a balanced nutritional profile of carbohydrates, proteins, lipids, minerals, vitamins, organic acids, chlorophylls, phenolic compounds, and carotenoids (Wang et al., 2021). Because of its nutritional qualities, kiwifruit has been considered as the “king of fruits” for centuries (Huang et al., 2013).

Kiwifruit is believed to have originated in the Yangzi and Pearl Rivers and mountainous ranges in China (Huang et al., 2013). Historical records of kiwifruit vine cultivars trace back to the 7^th^ century A.D. during the Tang Dynasty. In the early 20^th^ century, kiwifruit cultivation spread from China to New Zealand, where the commercial planting of this fruit was initiated (Huang and Ferguson, 2007). Its commercial cultivation has since spread to other significant producing countries. China is now the world’s largest kiwifruit producer and presented an annual production of approximately 2.20 million tons in 2019, followed by New Zealand (0.56 million tons), Italy (0.52 million tons), Iran (0.34 million tons), and Greece (0.29 million tons), with these countries accounting for 50.52, 12.84, 12.06, 7.92, and 6.57% of the global annual production (4.35 million tons), respectively. However, in terms of yield per unit area, China (120,325 hg/ha) is much lower than the world average (161,764 hg/ha) and ranks at approximately the 20^th^ among the kiwifruit producing countries, and it lags behind that of New Zealand (374,073 hg/ha) and Greece (277,804 hg/ha) (FAOSTAT, 2019). This phenomenon has substantially restricted the economic output. Thus, improving the yield per unit area is of critical importance for the current kiwifruit industry in China (Fang and Zhong, 2019; Qi et al., 2020).

Yield formation can be controlled by the integration of various factors, such as genotypes, environmental conditions, and cultural practices. Kiwifruit vines are best trained upward on trellises because they can become unruly within one season and require sturdy support to protect the vines from tangling and shadowing one another. The tangled and shaded vines yield less fruit. T-bar and overhead pergola structures are two main types of support trellis systems commonly used in commercial kiwifruit production (Costa and Biasi, 1991). T-bar trellis is easier to build, less labor-intensive, and better for bee pollination, and it reduces the risk of contracting Botrytis compared with the pergola trellis (Strik and Cahn, 2000). However, the pergola trellis may increase productivity and reduce the vulnerability of resulting fruit to wind damage. Additionally, once the complete pergola canopy is in place, the shade slows the growth of weeds (Hartmann, 2018).

Kiwifruit has become a mainstream fruit crop and advantageous characteristic industry in Sichuan Province, where the traditional overhead pergolas trellis (OPT) is used. In our previous study, we developed an improved overhead pergolas trellis, the umbrella-shaped trellis (UST) system, for Sichuan kiwifruit production. Surprisingly, UST has been found to be effective at enhancing kiwifruit yield based on more than 5 years of observations by local growers. However, the mechanisms underlying the high yield of the UST are still unclear.

To study the mechanisms of high yield of the UST, a two-consecutive-years comparative study of the effects of the UST and OPT on kiwifruit yield was performed in this work. The newly developed cultivar, ‘Donghong’ (DH) kiwifruit (*A. chinensis*), which descended from the F1 offspring of open-pollinated ‘Hongyang’ (*A. chinensis*) kiwifruit (Zhong et al., 2016), was selected as experimental material. DH is now the second most popular and widely cultivated red-fleshed kiwifruit in China (Huang et al., 2022). To understand mechanisms underlying the high yield of the UST, the shoot growth, photosynthetic capacity, flower bud differentiation, and fruit quality and yield were evaluated. Elucidation of the regulatory mechanism will provide a scientific basis for the efficient increase in kiwifruit production.

## Materials and methods

### Plant materials and experimental site

An experimental trial was conducted over two consecutive years in 2020 and 2021 at a commercial kiwifruit orchard of the Xian Nong Fen Xiang Organic Agriculture Development Co. Ltd., which is located at Pujiang County (30°27′N, 103°42′E) of Sichuan Province, China. The climate was a subtropical monsoon climate with a wild summer and mild winter, and annual average rainfall, sunshine and temperature of 1,117.3 mm, 1,107.9 h and 16.3°C, respectively. Five-year-old plants (grafted onto wild *A. deliciosa*) bearing kiwifruit with uniform health, size, and vigor were used. The planting density was 667 vines/ha, with 3.0 m spacing between plants and 6.0 m spacing between rows. The row orientation was from north to south. All plants were subjected to identical standard orchard management practices for DH kiwifruit in this area throughout the experiment, including winter pruning, pest and pathogen control, basal fertilization, and irrigation.

### Experimental design and treatment

**Figure 1:**
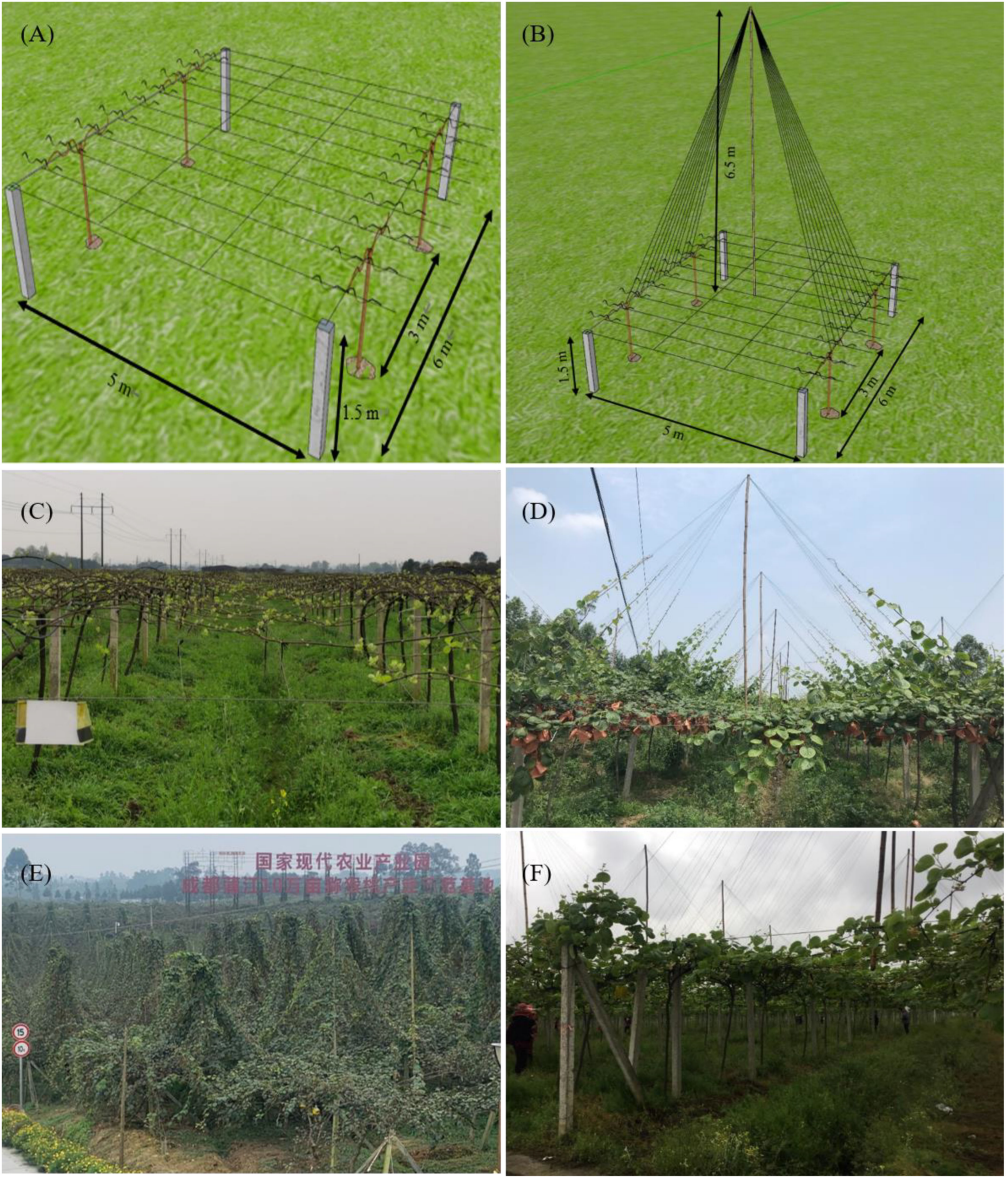
Schematic representations and field phenotypic characteristics of the traditional overhead pergolas trellis (OPT) and umbrella-shaped trellis (UST). **(A)** Schematic diagram illustrating the cane configuration (tree architecture) of the OPT, **(B)** schematic diagram illustrating cane configuration (tree architecture) of the UST, **(C)** field performance of the OPT, and **(D–F)** field performances of the UST.

The OPT was constructed with a number of vertical posts with a horizontal beam attached at the top that runs continuously between and perpendicular to the rows (**Figure 1A**). As an improved OPT system, the UST included a bamboo pole stands at the top center of four vertical posts with wires attached to the center wires of the OPT and the top of the bamboo pole (**Figure 1B**). The detailed parameters are shown in the schematic diagrams (**Figures 1A, B**) drawn using SketchUp software (https://www.sketchup.com). Their phenotypic characteristics in the field are shown in **Figures 1C–F**. The trees were trained with a straight vertical trunk approximately 1.5 m high and two central leader vines. The horizontal central leader produced the fruiting framework, which was made up primarily of one-year-old canes (**Figure 1C**). The vegetative canes (lateral branches) ran along the wires to the top, thus forming a canopy that resembled an “umbrella”. The fruited canes during the previous season as well as any dead, diseased, or twisted canes were pruned during winter, while those canes on the “umbrella” canopy were pulled down to horizontal in the following year to continue growing into flowering and fruiting canes (**Figures 1D–F**). Each treatment had three biological replicates, which each consisted of at least ten canes that were randomly chosen and tagged. Each flower on the tagged canes received supplementary pollination during blooming by being dusted with commercial bee-collected male pollen.

### Chemicals and reagents

High performance liquid chromatography (HPLC)-grade regents, including zeatin riboside (ZR), auxin (IAA), abscisic acid (ABA), and gibberellin (GA), were purchased from Sigma–Aldrich (St Louis, MO, USA). Aqueous solutions were prepared using ultra-purified water (18.2 MΩ cm) from a Milli-Q gradient water purification system (Millipore Corporation, Bedford, MA).

### Determination of cane length and diameter, bud number, and percentages of budbreak and flower bud

The length of current-year canes with diameters of 6 ~ 8 mm (slender), 8 ~ 10 mm (medium), > 10 mm (thick) were recorded on June 28, July 11, July 24, August 10, and August 24, 2020, while those with diameters less than 6 mm were not measured because they were heavily curled and too thin. The cane thickness was measured using calipers. The numbers of buds, budbreak, and flower buds on one-year-old slender, medium, and thick canes were counted at the flowering season (April 9, 2021). The corresponding proportion of budbreak and flowering shoots were calculated.

### Determination of leaf gas exchange parameters and photosynthesis pigment contents

The gas-exchange parameters included the net photosynthetic rate (*P_n_*), stomatal conductance (*Gs*), intercellular CO_2_ concentration (*Ci*), and transpiration rate (*Tr*). The measurements performed on the completely expanded leaves of the third to fifth node of canes on the UST upper canopy, fruiting canopy, and OPT fruiting canopy at the same time (09:00 am ~ 11:00 am at the end of June and the beginning of July of 2021) using a Li-6400XT portable photosynthesis system (Li-COR Inc., Lincoln, NE, USA). The upper canopy doesn’t bear fruit in UST system. The photosynthetic pigments, including chlorophyll a (Chl a), chlorophyll b (Chl b), and total carotenoids (TC), were extracted using fresh mature leaves (30 mg) in 95% ethanol (v/v, 3 ml). The photosynthetic pigment contents were determined using equations developed in a previous report (Molnárová and Fargašová, 2009).

### Determination of endogenous hormones using HPLC coupled with ultraviolet (UV) detection

Extraction and quantification of the endogenous hormones, including ZR, IAA, ABA, and GA, in the buds were performed using HPLC coupled with UV detection following our previously published protocol (Liang et al., 2019) with minor modifications. Briefly, samples were ground to a fine powder in liquid nitrogen. Then, 300 mg of powder was transferred into 2.0 mL conical-bottom plastic tubes and 1.5 mL of ice-cold extraction solvent containing 80% aqueous methanol and 0.5% formic acid was added. The samples were extracted by sonication for 5 min in an ultrasonic bath (Branson Ultrasonic Corporation, Connecticut, USA), cooled with ice water, and then agitated for 15 min at 4°C in a refrigerator using a rotator at 50 rpm. The homogenates were centrifuged (12,000 rpm, 10 min, 4°C) and the resultant supernatant was carefully collected into a clean amber vial. This extraction procedure was performed in triplicate. Thereafter, the combined supernatants were evaporated to water phase under vacuum at 37°C using an N-EVAP analytical evaporator (Organomation Associates Inc., South Berlin, MA, USA). The residue was dissolved in 200 μL of 30% (v/v) acetonitrile. Each extract was purified *via* a 0.22 μm porosity polytetrafluoroethylene membrane prior to injection in the HPLC system.

The HPLC system consisted of an Agilent 1260 HPLC instrument and a diode array detector (Agilent, Santa Clara, CA, USA). Chromatographic separation was achieved using a ZORBAX StableBond C_18_ column (inner diameter × length: 4.6 mm × 150 mm; particle size: 5 μm) (Agilent). Water containing 45% methanol and 0.6% acetic acid was used as the mobile phase, with a flow velocity of 1.0 mL min^-1^. The column temperature over was maintained at 35°C, and the injection volume was 20 μL. Peaks were detected using a UV-visible detector at 270, 218, 200, and 270 nm for ZR, IAA, ABA, and GA_3_, respectively. The peak area of each endogenous hormone was quantified by comparing with the corresponding peak area of authentic standard containing known concentration.

### Determination of carbon (C) to nitrogen (N) ratio of bud

The anthrone colorimetric method was employed to measure the content of the soluble sugar and starch (Dubois et al., 1956). A Braford assay utilizing Coomassie brilliant blue G-250 was used to quantify the content of soluble protein (Bradford, 1976). The C/N ratio was defined as the sum contents of soluble sugar and starch to the sum content of soluble protein.

### Determination of the physicochemical parameters of fruit quality and yield

One hundred fruit samples from each treatment were randomly harvested at the end of August, which corresponds to the commercial ripening stage based on the total solid soluble (TSS) content > 7.5°Brix. These samples were randomly divided into five biological replicates. The single fruit weight was obtained from an average of 20 fruits of each biological replicate. The fruit shape index was calculated by dividing the fruit vertical length with transverse diameter. Dry weight was defined as the weight recorded after drying fruity slices at 60°C. TSS was determined using a digital refractometer (Model PAL1 0~53%; Atago Co., Ltd., Tokyo, Japan), and the corresponding result was expressed as °Brix. The soluble sugar content (SSC) was determined using the anthrone colorimetric method. Titratable acidity (TA, also called total acidity) was determined by standard acid-base titration methods following (Horwitz and Latimer, 2006), and the result was expressed as percentage of citric acid.

### Data processing and statistical analysis

All data are shown as the means ± SD of at least three replicates. One-way analysis of variance (ANOVA) and Tukey’s honestly significant difference (HSD) test (*P* < 0.05) were applied to examine the statistical significance of responses of kiwifruit to the OPT and UST systems. All statistical analyses were conducted with Minitab software v19.0 (Minitab, State College, PA, USA).

## Results

### Effect of the OPT and UST on the yield and quality of DH kiwifruit

The schematic representations and field phenotypic characteristics of the traditional OPT and UST systems were shown in **Figure 1**. The effects of the OPT and UST systems on DH kiwifruit yield and its components (fruit weight, fruit number per fruiting cane, and fruiting cane number per plant) were evaluated (**Table 1**). The estimated yield (3528.6 kg/667 m^2^) in the UST-treated group was 264.81% greater than that in the OPT control group (1332.5 kg/667 m^2^). The single kiwifruit weight or fruiting cane number per plant was not affected (*P* > 0.05) by the treatments. The average kiwifruit number per fruiting cane in the OPT control group was 36.7 ± 1.15, while it significantly increased *(P* < 0.05) by 70.03% in the UST-treated group (62.4 ± 6.80). Changes in the estimated yield paralleled those of the average kiwifruit number per fruiting cane, although even greater differences were observed due to the minor increment in fruiting cane number per plant under the UST treatment (**Table 1**).

**Table 1:** Effects of the OPT and UST on the estimated yield of Donghong kiwifruit.

Treatment	Single fruit weight (g)	Fruit number per fruiting cane	Fruiting cane number per plant	Plant number per 667 m^2^	Estimated yield (kg/667 m^2^)
OPT	79.09 ± 1.47a	36.65 ± 1.15b	10.33 ± 0.57a	44.5	1332.5
UST	80.91 ± 1.52a	77.35 ± 6.80a	12.67 ± 0.58a	44.5	3528.6

Data were expressed as means ± SD. Different letters within each column indicate significant differences (P < 0.05).

The extrinsic quality (fruit shape index) was similar (*P* > 0.05) in DH kiwifruit from OPT- (control) and UST-treated trees at both the harvest and ripening stages. At harvest, DH kiwifruit grown on the UST had significantly lower (*P* < 0.05) TA content (1.36 ± 0.03c g 100 g^-1^) than that in the control treatment (1.58 ± 0.02b g 100 g^-1^). The increases in TSS and SSC observed in all samples were due to fruit ripening after storage, although significant differences were not observed (*P* > 0.05) between those of the OPT- and UST-treated DH kiwifruit at both the harvest and ripening stages. The TSS/TA levels increased after storage in all treatments. At the ripening stage, the UST-treated DH kiwifruit showed significantly higher (*P* < 0.05) TSS/TA (7.54 ± 0.06a) compared to the control (6.40 ± 0.01b). Dry matter slightly decreased in all groups during storage, although significant differences were not observed between the different samples (**Table 2**).

**Table 2:** Extrinsic and intrinsic quality parameters in the OPT and UST-treated Donghong kiwifruit at harvest and after storage (ripening).

Parameter	Harvest		Ripening	
	OPT	UST	OPT	UST
fruit shape index	1.30 ± 0.07a	1.32 ± 0.09a	1.30 ± 0.07a	1.32 ± 0.09a
total soluble solids (°Brix)	7.23 ± 0.06b	7.57 ± 0.12b	13.25 ± 0.01a	13.73 ± 0.06a
soluble sugar content (%)	5.26 ± 0.17b	5.84 ± 0.15b	6.37 ± 0.10a	6.89 ± 0.07a
titratable acidity (%)	1.58 ± 0.02b	1.36 ± 0.03c	2.07 ± 0.06a	1.82 ± 0.12b
total soluble solids/titratable acidity	5.24 ± 0.03c	5.57 ± 0.08c	6.40 ± 0.01b	7.54 ± 0.06a
dry matter (%)	16.24 ± 0.004a	16.35 ± 0.002a	15.37 ± 0.006a	15.23 ± 0.001a

Data were expressed as means± SD. Different letters within each row indicate significant differences (P < 0.05).

### Effect of OPT and UST on cane and bud growth of DH kiwifruit

The total one-year-old cane length of the UST-treated trees during the whole period was significantly higher (*P* < 0.05) than that of the OPT control trees. Similar trends in the slender and medium canes were observed, and the maximum values of 145.11 ± 2.17 cm and 104.69 ± 3.55 cm were found on June 28 and August 24 in the UST-treated trees and controls, respectively, which accounted for 62% and 38% of their total one-year-old cane length, respectively. However, an opposite trend was observed in the thick canes throughout the whole period, with a significantly lower (*P* < 0.05) maximum value (52.35 ± 1.57 mm, 19%, recorded on August 24) of the UST-treated trees compared with the control (115.08 ± 2.72 mm, 56%) (**Table 3**).

**Table 3:** Effects of the OPT and UST on one-year-old cane length of Donghong kiwifruit.

Date	Treatment	Cane length (cm)				Ratio
		Total length	Slender canes with 6 ~ 8 mm in diameter	Medium canes with 8 ~ 10 mm in diameter	Thick canes with > 10 mm in diameter	
June 28	OPT	171.05 ± 4.18f	51.32 ± 1.35cd	51.32 ± 1.55c	68.42 ± 1.28c	30:30:40
	UST	245.75 ± 5.49c	145.11 ± 2.17a	88.94 ± 2.73b	11.70 ± 0.59g	59:36:05
July 11	OPT	194.73 ± 5.56e	59.50 ± 1.98c	59.50 ± 1.69c	75.73 ± 1.89bc	31:31:38
	UST	236.50 ± 4.80c	134.81 ± 2.33ab	80.41 ± 1.92b	21.29 ± 0.55f	57:34:09
July 24	OPT	182.43 ± 4.11e	49.26 ± 1.56d	47.43 ± 1.08d	85.74 ± 1.47b	27:26:47
	UST	246.30 ± 4.28b	135.47 ± 2.25ab	83.74 ± 1.52d	27.09 ± 0.51ef	55:34:11
August 10	OPT	203.40 ± 6.14d	46.78 ± 1.61de	48.84 ± 1.77d	107.80 ± 2.76a	23:24:53
	UST	251.77 ± 7.25b	118.33 ± 3.95b	103.23 ± 2.61a	30.21 ± 0.69e	47:41:12
August 24	OPT	207.56 ± 5.28d	42.16 ± 1.59e	49.32 ± 0.97d	115.08 ± 2.72a	21:24:55
	UST	281.02 ± 10.33a	123.98 ± 5.21b	104.69 ± 3.55a	52.35 ± 1.57d	44:37:19

Data were expressed as means ± SD. Different letters within each column indicate significant differences (P < 0.05).

The bud number per meter on canes with different diameters of the UST-treated group was significantly higher (*P* < 0.05) than that of the control. The highest amounts were recorded at August 24 (18.29 ± 1.92), July 24 (19.95 ± 2.07), and August 10 (18.53 ± 1.65) in the slender, medium, and thick canes of the UST-treated trees, respectively, which were 1.25, 1.29, and 1.21 times higher than those of the OPT control group (**Table 4**).

**Table 4:** Effects of OPT and UST on bud number per meter of Donghong kiwifruit.

Date	Treatment	Slender canes with 6 ~ 8 mm in diameter	Medium canes with 8 ~ 10 mm in diameter	Thick canes with > 10 mm in diameter
June 28	OPT	11.21 ± 0.79f	12.53 ± 0.83f	13.23 ± 1.71e
	UST	14.01 ± 1.05d	15.31 ± 1.51c	15.85 ± 1.85bc
July 11	OPT	11.52 ± 0.83f	13.82 ± 1.27de	13.03 ± 1.62e
	UST	13.52 ± 0.97e	14.21 ± 1.35d	16.59 ± 1.56b
July 24	OPT	15.13 ± 1.25c	15.52 ± 1.69c	14.26 ± 1.72d
	UST	16.73 ± 1.92b	19.95 ± 2.07a	18.38 ± 1.93a
August 10	OPT	15.33 ± 1.37c	13.25 ± 1.17e	15.32 ± 1.77c
	UST	15.41 ± 1.29c	15.11 ± 1.39c	18.53 ± 1.65a
August 24	OPT	14.58 ± 1.43d	15.18 ± 1.83c	15.91 ± 1.27bc
	UST	18.29 ± 1.92a	17.39 ± 1.92b	18.37 ± 1.95a

Data were expressed as means ± SD. Different letters within each column indicate significant differences (P < 0.05).

UST-treated trees had significantly higher (*P* < 0.05) total lengths of slender canes (156.33 ± 2.31 mm) and medium canes (115.72 ± 2.69 mm), bud number per fruiting cane (60.4), budbreak number (31.83), and flower bud number (31.32) on the fruiting cane, which were 2.39, 2.34, 1.78, 1.94 and 2.00 times higher than that of the OPT control group. No significant differences in these parameters for the thick canes were observed between trees treated with the OPT or UST system (**Table 5**).

**Table 5:** Effects of the OPT and UST on fruiting canes of Donghong kiwifruit.

Fruiting canes with different diameters	Treatment	Total fruiting cane length (cm)	Bud number	Budbreak number	Budbreak rate (%)	Flower bud number	Flower bud rate (%)
slender canes with 6 ~ 8 mm in diameter	OPT	65.37 ± 1.27e	9.67 ± 0.13c	5.78 ± 0.13c	59.73 ± 0.31a	5.74 ± 0.15c	59.38 ± 0.27a
	UST	156.33 ± 2.31a	23.50 ± 0.27a	14.31 ± 0.10a	60.89 ± 0.35a	14.21 ± 0.13c	60.47 ± 0.22a
medium canes with 8 ~ 10 mm in diameter	OPT	49.32 ± 1.15f	7.67 ± 0.15d	4.10 ± 0.09d	53.48 ± 0.27b	4.04 ± 0.02d	52.69 ± 0.19b
	UST	115.72 ± 2.69b	20.67 ± 0.21b	11.30 ± 0.17b	54.67 ± 0.41b	11.16 ± 0.05b	53.99 ± 0.21b
thick canes with > 10 mm in diameter	OPT	103.27 ± 1.58c	16.57 ± 0.11d	6.51 ± 0.03e	39.26 ± 0.37c	5.89 ± 0.01e	35.56 ± 0.11c
	UST	82.51 ± 1.30d	16.23 ± 0.11d	6.22 ± 0.11e	38.33 ± 0.15c	5.95 ± 0.01e	36.63 ± 0.13c

Data were expressed as means ± SD. Different letters within each column indicate significant differences (P < 0.05).

### Effect of the OPT and UST on the leaf gas exchange parameters and photosynthesis pigment content of DH kiwifruit

**Figure 2:**
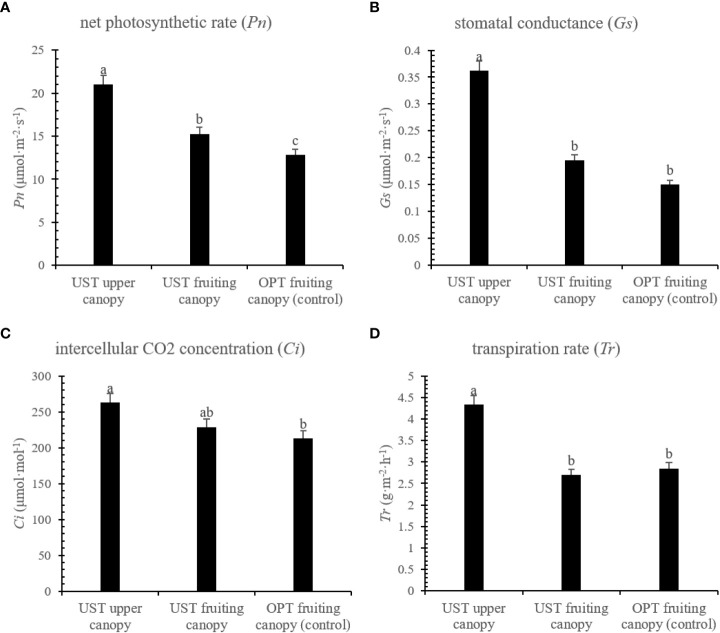
Effects of the OPT and UST on **(A)** net photosynthetic rate (*Pn*), **(B)** stomatal conductance (*Gs*), **(C)** intercellular CO_2_ (*Ci*), and **(D)** transpiration rate (*Tr*) of Donghong kiwifruit. The lower-case letters on the bars indicate significant differences (*P* < 0.05).

The UST system significantly (*P* < 0.05) increased the *Pn*, *Gs*, *Ci*, and *Tr* of the upper canopy by 64.25, 141.80, 23.47, and 51.93% compared with that observed for the OPT fruiting canopy. There was no significant (*P* > 0.05) change in the leaf gas exchange parameters of the UST fruiting canopy compared to the OPT fruiting canopy except for *Pn* (**Figure 2**).

**Figure 3:**
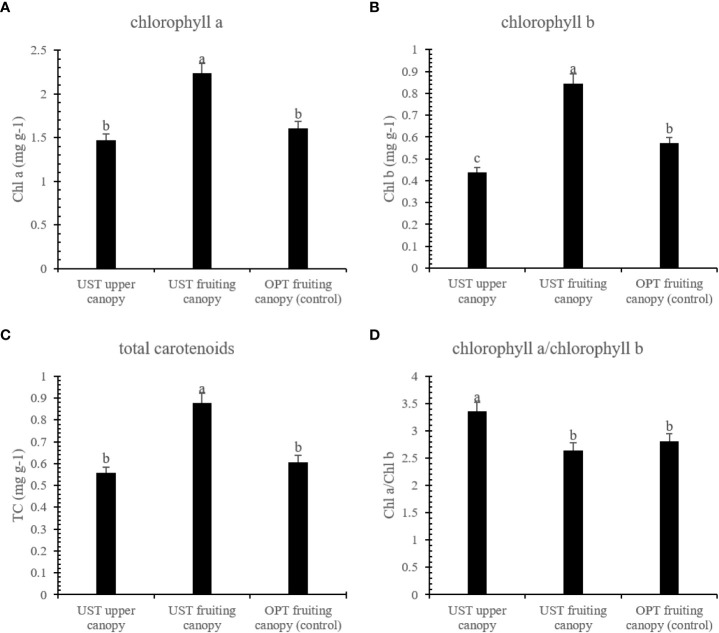
Effects of the OPT and UST on **(A)** chlorophyll a (Chl a), **(B)** chlorophyll b (Chl b), **(C)** total carotenoid (TC), and **(D)** chlorophyll a/chlorophyll b of Donghong kiwifruit. The lower-case letters on the bars indicate significant differences (*P* < 0.05).

As shown in **Figure 3**, the UST system enhanced the Chl a, Chl b, and TC contents of the fruiting canopy by 39.38, 48.11, and 44.63%, respectively, compared to those of the OPT fruiting canopy, while the Chl a, Chl b, and TC contents of the UST upper canopy was lower than those of the OPT fruiting canopy (**Figure 3**).

### Effect of the UST on bud endogenous hormones of DH kiwifruit

**Figure 4:**
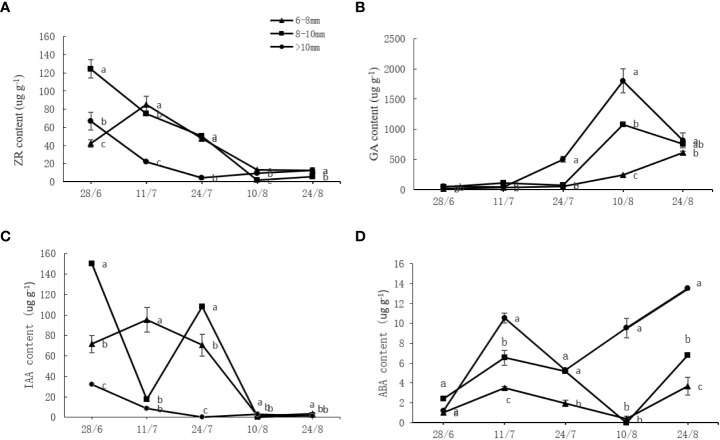
Effect of the UST on **(A)** zeatin riboside (ZR), **(B)** auxin (IAA), **(C)** abscisic acid (ABA), and **(D)** gibberellin (GA) content in buds of the upper canopy. The lower-case letters indicate significant differences (*P* < 0.05).

Different endogenous hormones underwent a series of changes throughout the measurement period. As shown in **Figure 4**, the endogenous ZR and IAA contents showed a declining trend while the GA content increased over time. The ABA content changes were irregular and initially increased, peaking on July 11 before sharply decreasing and further increasing. The ZR and IAA contents of the slender and medium canes were significantly higher (*P* < 0.05) than those of the thick canes at most time points, whereas the GA and ABA contents of the slender and medium canes were significantly lower (*P* < 0.05) than those of the thick canes (**Figure 4**).

**Figure 5:**
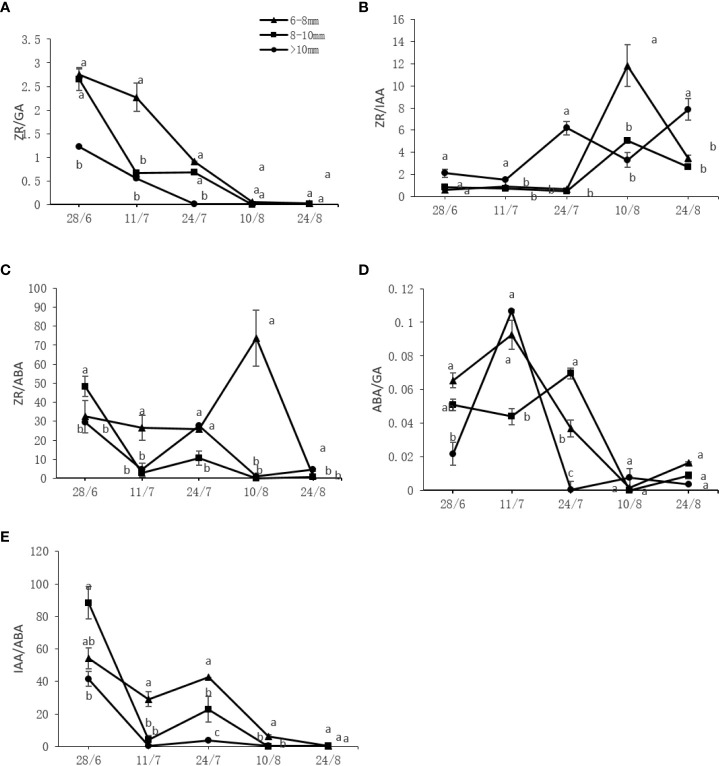
Effect of the UST on the **(A)** zeatin riboside (ZR)/gibberellin (GA), **(B)** ZR/auxin (IAA), **(C)** ZR/abscisic acid (ABA), **(D)** ABA/GA, and **(E)** IAA/ABA ratios in the bud on upper canopy. The lower-case letters indicate significant differences (*P* < 0.05).

The variation tendencies of the ratios of different endogenous hormones are shown in **Figure 5**. Overall, ZA/GA, ZA/ABA, and IAA/IBA roughly decreased with a similar pattern and timing but to different extents, while ZR/IAA and ABA/GA initially increased and then decreased. Significantly higher (*P* < 0.05) levels of ZR/GA, ZR/ABA, ABA/GA, and IAA/ABA were found on the slender and medium canes compared to the thick canes. For ZR/IAA, the opposite behavior was observed, with thick canes containing significantly higher (*P* < 0.05) levels of ZR/IAA compared to the slender and medium canes (**Figure 5**).

### Effect of the UST on the C/N ratio of DH kiwifruit

**Figure 6:**
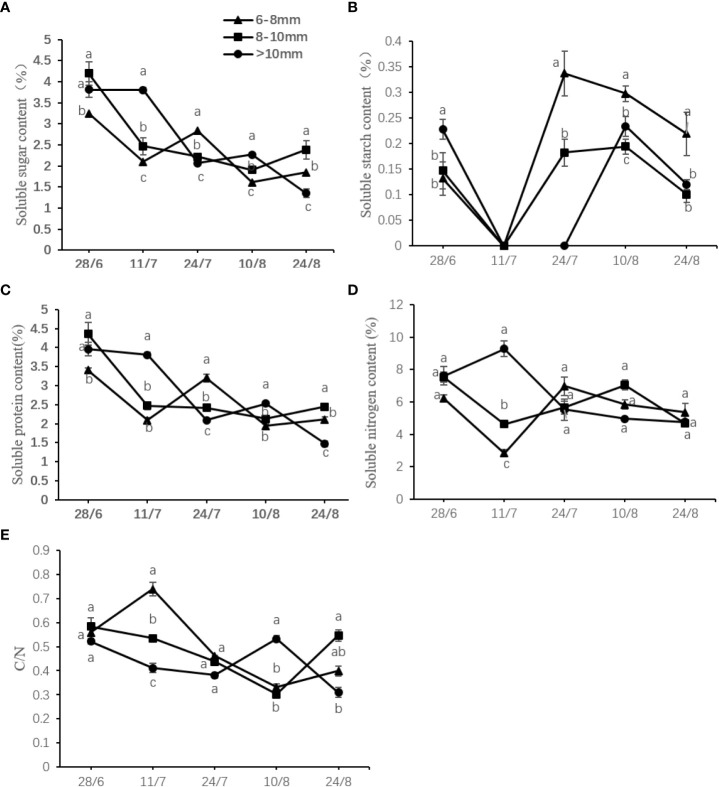
Effect of the UST on the **(A)** soluble sugar content, **(B)** soluble starch content, **(C)** soluble carbon content, **(D)** soluble protein content, and **(E)** C/N ratio in the bud on upper canopy. The lower-case letters represented significant difference (*P* < 0.05).

The C/N ratio showed decreasing trends similar to that of the soluble sugar, soluble carbon, and soluble nitrogen contents. The soluble starch content peaked transiently at the initial measurement period, declined rapidly to undetectable levels, then increased over the time course, and gradually decreased thereafter. On July 11, the C/N ratio of slender canes reached the maximum value of 0.74 at July 11, which was 1.38 and 1.80 times higher than that of the medium and thick canes, respectively (**Figure 6**).

## Discussion

Kiwifruit has been renowned as the “king of fruits” due to its nutritional characteristics (Huang et al., 2013). The present global production of kiwifruit stands at 4.35 million tons, with China accounting for more than half of the total. However, in terms of yield per unit area, China is much lower than the global average, placing roughly 20^th^ among kiwifruit producing countries (FAOSTAT, 2019). Improving the yield per unit area is a more profitable approach for the whole kiwifruit industry. Here, we consider the potential of the UST system to address yield challenges in our kiwifruit industry. Surprisingly, the estimated yield of the UST treatment is more than two times higher than that of the traditional OPT. In addition, significant differences were observed in the internal fruit quality. These outcomes of this study may contribute to the sustainability of the kiwifruit industry.

### Vegetative growth of one-year-old canes

In its natural habitat, the kiwifruit plant is a fast-growing climbing vine that needs to be properly trellised under for commercial production (Costa and Biasi, 1991; Cieslak et al., 2011). In this study, UST system significantly (*P* < 0.05) promoted the vegetative growth of slender and medium canes with diameters of 6 ~ 10 mm, but inhibited the vegetative growth of thick canes with diameters > 10 mm (**Table 3**). The kiwifruit plant presents rapid growth and large biomass and is not self-supporting. Its cane weight increases as it grows, resulting in cane bending, which hinders apical dominance by stimulating the growth of lateral and axillary buds. A plausible explanation for the observed significantly vigorous growth of one-year-old canes is that when the canes were treated upward on the UST system, the cane weight was supported, the scrambling vegetative growth was managed, and the preferred apical dominance of one-year-old fruiting canes was maintained (Barbier et al., 2017).

### Leaf photosynthesis

The upper umbrella-shaped canopy of the UST served as a natural shading condition for the lower fruiting canopy (**Figure 1**). In previous studies, proper shading has been shown to positively affect the kiwifruit yield and quality and alleviate the high temperature and intense sunlight stresses (Biasi et al., 1995; He et al., 2007). In this study, the UST system significantly (*P* < 0.05) increased the leaf gas exchange parameters of the upper canopy (**Figure 2**). An intriguing interpretation is that the UST system allows the canopy to better exploit both the upper and lower spaces with sufficient light and ventilation. Significant (*P* > 0.05) changes were not observed between the UST and OPT fruiting canopies (**Figure 2**), which is consistent with previous studies (Rosenqvist et al., 1991; He et al., 2007). One potential explanation is that the shading function of the upper canopy to the lower fruiting canopy is still within the range of proper shading and the fruiting canopy is still capable of capturing sufficient sunlight for photosynthesis under summer conditions (Rosenqvist et al., 1991).

Photosynthesis is a main process in plant development and physiology that is closely linked to crop yield. Leaf chlorophyll molecules are essential pigments that determine photosynthesis rates (Croft et al., 2017). In this study, the UST treatment had positive effects on the accumulation of Chl a, Cl b, and TC in the fruiting canopy, while negative effects on these parameters were observed in the upper canopy (**Figure 3**). The excessive sunlight stress can be especially severe in summer, when a lack of available water combined with high temperatures severely limits the foliar ability to use radiant energy for photosynthesis. The intense sunlight in summer might promote photosynthetic pigment degradation (Miller et al., 2010; Pinheiro and Chaves, 2011; Flexas et al., 2014). With help of the upper canopy architecture, a relatively weak light condition was generated in the lower fruiting canopy, which triggered the plants to synthesize more photosynthetic pigments to maintain efficient light harvesting.

### Endogenous hormones, carbon-nitrogen ratio, and flower bud differentiation

The UST had a significant effect on one-year-old fruiting cane length (**Table 3**), bud number per meter (**Table 4**), budbreak number, and flower bud number (**Table 5**). The most productive zone was on the fruiting canes with a diameter of 6~10 mm (**Tables 1**-**5**). Flower bud differentiation is a critical step in the plant fruiting process, and endogenous hormones are key elements that contribute to flower bud differentiation (Sandoval-Oliveros et al., 2017; He et al., 2018); however, their effects on flower differentiation vary depending on the plant species. For example, GA has a promoting effect on flower formation of long-day and biennial plants (Mutasa-Göttgens and Hedden, 2009), but an inhibitory effect on *Arabidopsis thaliana* (Yamaguchi et al., 2014). In this study, the slender and medium fruiting canes on UST-treated trees with significantly higher (*P* < 0.05) budbreak and flower bud numbers (**Table 5**) contained significantly higher (*P* < 0.05) levels of ZR and IAA (**Figure 4**). The thick canes with significantly lower (*P* < 0.05) budbreak and flower bud numbers (**Table 5**), however, contained significantly higher (*P* < 0.05) levels of GA and ABA (**Figure 4**).

A single hormone cannot exert a substantial impact on plant flower bud differentiation. As a complex physiological and biochemical process, the bud differentiation is generally coordinated by various hormones and is greatly influenced by the content and ratio of hormones (Domagalska et al., 2010). In this study, the slender and medium fruiting canes on UST-treated trees with significantly higher (*P* < 0.05) budbreak and flower bud numbers (**Table 5**) contained significantly higher (*P* < 0.05) levels of ZR/GA, ZR/ABA, and ABA/GA (**Figure 5**). This suggests that high levels of ZR/GA, ZR/ABA, and ABA/GA ratios might be beneficial to flower bud differentiation of DH kiwifruit.

The C/N ratio is considered an indicator of plant growth and flower quality characteristics. High C/N ratios are capable of promoting reproductive growth, whereas low C/N ratios are thought to promote early vegetative growth or even inhibit flowering (Tsai and Chang, 2022). The results here (**Figure 6**) showed that a relatively high C/N ratio may promote the flower bud differentiation process of DH kiwifruit.

## Conclusions

The current kiwifruit yield per unit area in China is not keeping pace with its global production ranking. In this study, an UST system was developed with the aim of increasing kiwifruit yield. Surprisingly, UST-treated trees were twice as productive than traditional OPT-treated system. UST-treated trees also presented improved fruit quality. The UST system significantly (*P* < 0.05) promoted the vegetative growth of fruiting canes with diameters of 6 ~ 10 mm and had positive effects on the accumulation of chlorophyll and total carotenoid contents in the fruiting canopy. The high yield may also be due to the improvements in ZR and IAA contents and ZR/GA, ZR/ABA, and ABA/GA ratios. A high ratio of C/N might be beneficial to promoting the flower bud differentiation process.

## Data availability statement

The original contributions presented in the study are included in the article/supplementary material. Further inquiries can be directed to the corresponding authors.

## Author contributions

HX and DL conceived and designed the research. HD, YLi, KZ, CP, XT, TW, YLiang, ZH, and YLang performed the experiments. HD, YLi, JF, LL, JW, and XL analyzed the data. HD prepared and wrote the manuscript. HD, HX, and DL checked and revised the manuscript. All authors contributed to the article and approved the submitted version.

## References

[B1] Barbier F. F. Dun E. A. Beveridge C. A. (2017). Apical dominance. Curr. Biol. 27, R864–R865. 28898653 10.1016/j.cub.2017.05.024

[B2] Biasi R. P.J M. Costa G. (1995). Light influence kiwifruit(*Actinidia deliciosa*)quality. Acta Hortic. 379, 245–251.

[B3] Bradford M. M. (1976). A rapid and sensitive method for the quantitation of microgram quantities of protein utilizing the principle of protein–dye binding. Anal. Biochem. 72, 248–254. 942051 10.1016/0003-2697(76)90527-3

[B4] Cieslak M. Seleznyova A. N. Hanan J. (2011). A functionalstructural kiwifruit vine model integrating architecture, carbon dynamics and effects of the environment. Ann. Bot. 107, 747–764. 20855486 10.1093/aob/mcq180 PMC3077975

[B5] Costa G. Biasi R. (1991). Comparison of kiwifruit traininng systems. Acta Hortic. 297, 427–434.

[B6] Croft H. Chen J. M. Luo X. Bartlett P. Chen B. Staebler R. M. (2017). Leaf chlorophyll content as a proxy for leaf photosynthetic capacity. Glob. Change Biol. 23, 3513–3524. 10.1111/gcb.13599 27976452

[B7] Domagalska M. A. Sarnowska E. Nagy F. Davis S. J. (2010). Genetic analyses of interactions among gibberellin, abscisic acid, and brassinosteroids in the control of flowering time in arabidopsis thaliana. PloS One 5, e14012. 21103336 10.1371/journal.pone.0014012 PMC2984439

[B8] Dubois M. Gilles K. A. Hamilton J. K. Rebers P. A. Smith F. (1956). Colorimetric method for determination of sugars and related substances. Anal. Chem. 28, 350–356.

[B9] Fang J. Zhong C. (2019). Fruit scientific research in new China in the past 70 years: kiwifruit (In Chinese with English abtract). J. Fruit Sci. 36, 1352–1359.

[B10] FAOSTAT (2019) Top 10 kiwi fruit producing countries in the world - production and area under cultivation. Available at: https://numerical.co.in/numerons/collection/611a67171785141f7892d4af (Accessed September 26, 2022).

[B11] Ferguson A. R. (2016). “Botanical description,” in The kiwifruit genome, compendium of plant genomes. Eds. Testolin R. Huang H. Ferguson A. R.(Springer Switzerland), 1–14.

[B12] Flexas J. Diaz-Espejo A. Gago J. Gallé A. Galmés J. Gulías J.. (2014). Photosynthetic limitations in Mediterranean plants: A review. Environ. Exp. Bot. 103, 12–23.

[B13] Hartmann T. (2018) Establishing kiwifruit orchards in Texas. Available at: https://aggie-horticulture.tamu.edu/kiwifruit/content/fact_sheets/kiwifruit_orchards_in_texas.pdf (Accessed September 26, 2022).

[B14] He K. Wang Z. Wang R. (2007). Effects of overhead shading in summer on ecological factors and photosynthesis of kiwifruit orchard. J. Fruit Sci. 24, 616–619.

[B15] W. Horwitz G. W. Latimer (Eds.) (2006). Official methods of analysis of AOAC international. 18th edition (Gaithersburg, MD: AOAC International Gaithersburg).

[B16] He W. Chen Y. Gao M. Zhao Y. Xu Z. Cao P.. (2018). Transcriptome analysis of *Litsea cubeba* floral buds reveals the role of hormones and transcription factors in the differentiation process. G3 Genes Genomes Genet. 8, 1103–1114. 10.1534/g3.117.300481 PMC5873901 29487185

[B17] Huang H. W. Ferguson A. R. (2007). *Actinidia* in China: Natural diversity, phylogeographical evolution, interspecific gene flow and kiwifruit cultivar improvement. Acta Hortic. 753, 31–40.

[B18] Huang W. Wang Z. Zhang Q. Feng S. Burdon J. (2022). Maturity , ripening and quality of ‘ donghong ‘ kiwifruit evaluated by the kiwi-meter ^TM^ . Horticulture 8, 852.

[B19] Huang S. Ding J. Deng D. Tang W. Sun H. Liu D.. (2013). Draft genome of the kiwifruit *Actinidia* chinensis. Nat. Commun. 4, 2640. 24136039 10.1038/ncomms3640 PMC4089393

[B20] Liang D. Huang X. Shen Y. Shen T. Zhang H. Lin L.. (2019). Hydrogen cyanamide induces grape bud endodormancy release through carbohydrate metabolism and plant hormone signaling. BMC Genomics 20, 1034. 31888462 10.1186/s12864-019-6368-8 PMC6937986

[B21] Miller G. Suzuki N. Ciftci-Yilmaz S. Mittler R. (2010). Reactive oxygen species homeostasis and signalling during drought and salinity stresses. Plant Cell Environ. 33, 453–467. 19712065 10.1111/j.1365-3040.2009.02041.x

[B22] Molnárová M. Fargašová A. (2009). Se(IV) phytotoxicity for monocotyledonae cereals (*Hordeum vulgare* l., *Triticum aestivum* l.) and dicotyledonae crops (*Sinapis alba* l., *Brassica napus* l.). J. Hazard Mater. 172, 854–861. 19709809 10.1016/j.jhazmat.2009.07.096

[B23] Mutasa-Göttgens E. Hedden P. (2009). Gibberellin as a factor in floral regulatory networks. J. Exp. Bot. 60, 1979–1989. 19264752 10.1093/jxb/erp040

[B24] Pinheiro C. Chaves M. M. (2011). Photosynthesis and drought: Can we make metabolic connections from available data? J. Exp. Bot. 62, 869–882. 21172816 10.1093/jxb/erq340

[B25] Qi X. Guo D. Wang R. Zhong Y. Fang J. (2020). Development status and suggestions on Chinese kiwifruit industry (In Chinese with English abtract). J. Fruit Sci. 37, 754–763.

[B26] Rosenqvist E. Wingsle G. Ögren E. (1991). Photoinhibition of photosynthesis in intact willow leaves in response to moderate changes in light and temperature. Physiol. Plant 83, 390–396.

[B27] Sandoval-Oliveros R. Guevara-Olvera L. Beltrán J. P. Gómez-Mena C. Acosta-García G. (2017). Developmental landmarks during floral ontogeny of jalapeño chili pepper (*Capsicum annuum* l.) and the effect of gibberellin on ovary growth. Plant Reprod. 30, 119–129. 28840335 10.1007/s00497-017-0307-0

[B28] Strik B. Cahn H. (2000) Growing kiwifruit. Available at: http://content.libraries.wsu.edu/index.php/utils/getfile/collection/cahnrs-arch/id/499/filename/96136182432004_pnw507.pdf (Accessed September 26, 2022).

[B29] Tsai S. S. Chang Y. C. A. (2022). Plant maturity affects flowering ability and flower quality in phalaenopsis, focusing on their relationship to carbon-to-nitrogen ratio. HortScience 57, 191–196.

[B30] Wang S. Qiu Y. Zhu F. (2021). Kiwifruit (*Actinidia* spp.): A review of chemical diversity and biological activities. Food Chem. 350, 128469. 33485721 10.1016/j.foodchem.2020.128469

[B31] Yamaguchi N. Winter C. M. Wu M.-F. Kanno Y. Yamaguchi A. Seo M.. (2014). Gibberellin acts positively then negatively to control onset of flower formation in *Arabidopsis* . Science 344, 638–641. 24812402 10.1126/science.1250498

[B32] Zhong C. Han F. Li D. Liu Xi. Zhang Q. Jiang Z.. (2016). Breeding of red-fleshed kiwifruit cultivar. “Donghong.” J. Fruit Sci. 32, 1596–1599.

